# Supplementary material for: Patterns of Intron Gain and Loss in Fungi
Source: PLoS Biol. 2004 Nov 30;2(12):e422. doi: 10.1371/journal.pbio.0020422 (PMC532390; doi:10.1371/journal.pbio.0020422)
Supplement: Table S1 — Also available at http://genes.mit.edu/NielsenEtAl/. (4.3 MB ZIP). [file pbio.0020422.st001.zip › NielsenEtAl/html/473.html]

AN5873.1.NCU05296.1.MG07129.1.FG06158.1


```
 CLUSTAL W (1.82) Multiple Sequence Alignments - Introns Inserted


Sequence 1: NCU05296.1	895 aa
Sequence 2: FG06158.1	907 aa
Sequence 3: MG07129.1	951 aa
Sequence 4: AN5873.1	893 aa
Alignment Length: 996 aa
Number Identitical Residues: 374 aa
Alignment Score (without introns) 19304


MG07129.1 	MSSTAAR---SVAADKMKKANGAGLAADSTTRSGTSRSRMNSTANNINNNNNNNNNNNNG
NCU05296.1	----------------------MGSSATLAPTQSTSQTRPTSR----------TLVN---
FG06158.1 	MSSGAPRPSGRTSANDYRDERPRGSAANLKSDRSDPRKGPSPQPNFSSAHKRSASVNPRA
AN5873.1  	MNPPRPR----VASSRVADAEEAGLRGGTAPLQAENMDHRGSTSTQKSKVPREHVTS---
          	 ..  .      ::.   .    *  .   .  .       . ..  .    .   .   

MG07129.1 	NNPNDTSFRSERERHNARYKEASPQPDSQSNSQTSHGYAQTSHKRSASGNLRPVSKLEER
NCU05296.1	------DRRPAKVQVTTR--DPAMARSG-RNADPLS------------------------
FG06158.1 	TSRHDEERRFESRRVTERTFEAQLERLVPRTTSPER------------------------
AN5873.1  	------EKRTERVVIQTKEKMQGRTRNPVRESPSAG------------------------
          	      . *        :             : .                          

MG07129.1 	RTEKVKVTTRETVVSRTRSPDRRGVQPPHREQRGSDLGKHRVATELRPKDNRPEQPQT~V
NCU05296.1	-------AGSDTLKSRSRNVESRRPRGDSRAAE--------------QQPQ--PPPPA~P
FG06158.1 	-------AHRRNAASESRNT-SKAPKNDIPDAR--------------QSRAGAETPQV1P
AN5873.1  	-------NRGEGDKSRSRRANTAGPLSPN------------------LRKKEKETPDA1P
          	              *.:*  .                                  * .  

MG07129.1 	WEPEASLLPLTTAPLASRISIPPLASHQPAALQPRPLYELPLAAQEALILEDLLYVFMGI
NCU05296.1	WEPAATLLPHTSAPLASRVSIPPVASSAPQTLQPRPLAELSLNAQEAAMIEDLLFVFMGF
FG06158.1 	WNPEATLLPHTTAPLASRISIPPLASTIPQAPQPKPLGELSLELQEAAIVEDLLFVFMGY
AN5873.1  	WNPHASLMPYTTAPLASRVSVPPLSSTLPQSLQPKPLRDLSTDAQEKAILEDLLFVFMGF
          	*:* *:*:* *:******:*:**::*  * : **:** :*.   **  ::****:**** 

MG07129.1 	EGQYIRYAKVYNPNEERDRLTGPTFRILPGLDPSLQDLTASMLRMATHYSALMEFVDVQS
NCU05296.1	EGQYIRFAKGYNPYEERDRLSGPQWRILSGLDPSLLDLTQSMLRMASHYTALETFVDVQS
FG06158.1 	EGQYIRFAKGYNPNEERDRLSGPSFRTLPGLDPSLQDLTQSMLRMATYYVALEAFVDVQS
AN5873.1  	EGQYIQYQAQYDPTAEKDRLTGPAFQLPSGLDPTLRDLTRSMLKMATHYSALEAFVEVQS
          	*****::   *:*  *:***:** ::  .****:* *** ***:**::* **  **:***

MG07129.1 	RDEFGTVNHALCASIRKLLQDYLVLIAQLETQFLTDDGFTLQVLNIHLLPTSHMMLQLHG
NCU05296.1	REEFGAVNHALCASIRRYLHEYLVMVVQLETQFLNDENFTLQQLNVHILPSSHLMAHMYG
FG06158.1 	REEFGSVNHALCASIRKFLQDYLVMIAQLETQFLTSDTFTVHVLNIHTMSTSQMMLQLYS
AN5873.1  	RAEYGAVSHALCATIRKLLKDYLILVAQLETQLLNNPNFTLHVLHLHTMPTSQCLAQLYS
          	* *:*:*.*****:**: *::**:::.*****:*..  **:: *::* :.:*: : :::.

MG07129.1 	LAHELLKKNALLDDESDESSDDDDPNDYDKIVNQLREGGDIIPGNMGGKKISKGGAVLGI
NCU05296.1	LALELLKRNALLDDDSDESSDSSD-DDFDHIIEQLRDGGDLVPGNMTGKKICKGGVVLGL
FG06158.1 	LAHELLKKNALLDDETDESEDSGG-DDFENILETLRDGGELVPGNMTGKKICKGGVVLGL
AN5873.1  	LGQELLRRNGLLDQDIDESIDDFD--DVDNILEQLREGGDLIPGAMSSKKICKGGNVLRL
          	*. ***::*.***:: *** *. .  * ::*:: **:**:::** * .***.*** ** :

MG07129.1 	ITSRLDTMSGDPAARALLTTLLKDASRPYMAMLNEWLHHGAINDPHAEFLVKEQRSIRRD
NCU05296.1	ITKRLESLSGDPSARVLLTTLLRDASRPYMAMLNEWLHHGSINDPHSEFLIKEQKSIRRE
FG06158.1 	ITRRLESLSGDPSARALLTTLLRDASKPYMNMLNGWLHHGSIHDPHSEFLIKEQKSIRRE
AN5873.1  	LTERLATFSGDPTTKALLETLLRESSRPYMTMLNEWLHHGVIKDPHAEFLVKEQKWIKRE
          	:* ** ::****:::.** ***:::*:*** *** ***** *:***:***:***: *:*:

MG07129.1 	RLEQDYTDEYWERRYTIRDQDVPPQLQGLKDKVLLAGKYLNVVRECGGVDVSKQVKDVPL
NCU05296.1	RLEQDYTDEYWERRYTIRDHDVPPQLEAVKDKVLLAGKYLNVVRECGGVDVSRKMQDVPN
FG06158.1 	RLEQDYTDEYWERRYTIRDHDVPPQLEGVKDKVLLAGKYLNVVRECGGVDVSKVAKDVPA
AN5873.1  	KLEEDYTDEYWEKRYTIRDNEVPPQLDSVRDKVLLAGKYLNVVRECGGVDVSKAVKDVPK
          	:**:********:******::*****:.::**********************:  :*** 

MG07129.1 	TFDDPRFLDNVHNAYAHANESLMQLLLTAHSLPARLGSMKHYFFLDPSDYFSYFLELGAA
NCU05296.1	SFDDNRFLENISSAYAHANGSLMQLLLTTHELPARLRSLKHYFFLDPSDYFNYFLELGTS
FG06158.1 	SFDDNRFLENVNNAYAHANESLMQLLLTAHSLPARLRSLKHYFFLDPSDYFSYFLELGAS
AN5873.1  	TFDDPRFLENVNAAYTYANASLLNLLLTKNSLTTRFRSLKHYFFLDRSDFFSYFLELGAS
          	:*** ***:*:  **::** **::**** :.*.:*: *:******* **:*.******::

MG07129.1 	ELKRPVKSVNTSKLQSLLDLVLRQPGTVVSLDPFKEDVKVEMNEVPLIKSLQRVVNITGL
NCU05296.1	ELRKPVKVVNTGKLQSLLDLVLRQPGSIVCLDKFKEDVKVEMNEVTLLKSLQRVVNITGI
FG06158.1 	ELRKPVKSVNTGKLQSLLDLVLRQPGSLVSLDPFKEDVKVEMNETILTKALQRVVNITGI
AN5873.1  	ELRKPAKNVNESKLQSLLDLVLRQPGSIAAQDPFKEDVKVRMNKIGLTKWLMQVVSVSGI
          	**::*.* ** .**************::.. * *******.**:  * * * :**.::*:

MG07129.1 	DVG--ETFQPSTSMP---SETDKSAIGFNCLQLDYAVPFPVSLVISRKTVWRYQALFRYL
NCU05296.1	EQG--EALQLTSNQP---LEGDKNATGFTSLQLDYTVPFPVSLVISRKTIWRYQALFRYL
FG06158.1 	EQG--EALQPLANQP---VDNDKNAVGFTSLQLDYSVPFPVSLVISRKTVWRYQALFRYL
AN5873.1  	DQDNPEAALEKYQAPQAGSEDDKDIVGFDALELDYTVPFPLSLVISRKTVLRYQLIFRHL
          	: ...*:     . *.:. : **.  ** .*:***:****:********: *** :**:*

MG07129.1 	LSLRHLESLLSNHWQAHNQWALWTRRSSSHRIEVFKRRVLTLRARMLVFVQQLLYFCTA~
NCU05296.1	LSLRYLETQLSVTWYTHASGTAWTHRSSNRMLEIWKRRVWTLRARMLVFVQQLLYFCTA~
FG06158.1 	LSLRYLESQLSTTWQTQTRGVSWAHKGGSRKLEIWKRRVWTLRA--------------P1
AN5873.1  	LSLRHLESMLSSAWLDQSKILGWRHKSTDRRLELWKRRAWNLRAKMLVFIQQLLYFCTA~
          	****:**: **  *  :     * ::. .: :*::***. .***      ..    .:. 

MG07129.1 	EVIQPNWHKFMQSLEKKEG-------AKGLTRT----------VDELMQDHVGFLDTCLK
NCU05296.1	EVIEPNWQKLMNTLREIENNCAGVTTQDGTSTPRASSAKKPHTVDGLMQEHVDFLDTCLK
FG06158.1 	EVIEPNWNKFMSRLKTKDE---SLDTSLGVSTR---------TVDELMQDHVDFLDTCLK
AN5873.1  	EVVEPNWQSLMDRVNGTDAD-GSEVTVNGTKQVN-------RTVDELMQDHVDFLDTCLK
          	**::***:.:*. :.  : . ..  :  * .           :** ***:**.*******

MG07129.1 	ECMLTNSKLLK0INAKLMHTVLLFATYSR-WFNQELKKADPDLAGPTKPAGMSD---RDW
NCU05296.1	ECMLTNSKLLR0IHSKLMQTCSVFATYTN-WLSRELEKSDPDLTGNTKPPNMTDDQWRQF
FG06158.1 	ECMLTNSKLLR0IHSKLMQTCTIFAAYTN-WLTRELEKSDPDLSGTNKPSTMTADQWKRF
AN5873.1  	ECMLTQAKLLK0IHSKLLTCCTMFASWTAAPLGRALISADPGLSAN--PATSDG------
          	*****::***: *::**:    :**::: : : : * .:**.*:.   *.          

MG07129.1 	ALIQALPTESKSSSGG---------EKPRDNMFEILAKWEVNFSRHLHIMLD-~--SLNH
NCU05296.1	LVIRGQRQQQQSKDGDTNMENDKNPETRMNELFDVIRKWESNFSRHLQILLD-~--ALNH
FG06158.1 	QMTKAVQRGSTSAQGDSSGSSE--ADARIDNLFEIIRKWESNFSRHLQILLD-~--ALNH
AN5873.1  	---RGYDP------------------ARIGKLEDTLKRYEDHFGRHLRILMDR2RLHSNT
          	   :.                        .:: : : ::* :*.***:*::*      * 

MG07129.1 	YAATETVVLLSLCARLSAASEGIETPGAREPDND~ST-~---
NCU05296.1	YAATETVVLLSLCARLSTANQGTEYAGLRSEEDG~GHG~GGS
FG06158.1 	YAATETVVLLSLCARLSTANQGTEYAGLRQEEES~T--~---
AN5873.1  	VTAASYIHQTYVCAGSPENKQSTFDPTTFDGRQS0LFC1K--
          	 :*:. :    :**  .  .:.   .   .  :.
```
